# Supplementary material for: Compensatory traits can explain the concave cost function of purely sexual traits
Source: Ecol Evol. 2024 Jan 14;14(1):e10850. doi: 10.1002/ece3.10850 (PMC10788312; doi:10.1002/ece3.10850)
Supplement: Supplementary file 1 — Figures S1‐S4 [file ECE3-14-e10850-s002.docx]

**Figure S1.** An example of performance surface with a saddle (left panel) and an example of performance surface with a ridge (right panel), both of which are given by performance surface = 0.5*γ_11_**x_1_^2^* + β_1_**x_1_* + 0.5*γ_22_**x_2_^2^* + β_2_**x_2_* + γ_12_**x_1_***x_2_* + α with the condition, γ_12_^2^ > γ_11_*γ_22_ (former) and γ_12_^2^ = γ_11_*γ_22_ (latter). Darker background coloration indicates lower performance.

**Figure S2.** The “corrected” predictions for manipulation experiment of Evans and colleagues under the presence of compensatory traits. Note that two type of scenarios (B and C) cannot be distinguished. See text and Fig. 1 for detailed explanations (also see Figs. 3 & S3).

**Figure S3.**  An example of hypothetical performance surface in relation to ornament and phenotypic expression of compensatory traits (upper panel). Darker background coloration indicates lower performance (note that the lightest background located near the current state, shown as a filled circle, in this example). Black arrow indicates hypothetical evolutionary pathway to the current state. Light blue arrow indicates phenotypic change when researchers experimentally reduced ornament expression (in which compensatory traits remain unchanged). Lower panel shows performance cost measurement (which is here denoted as the best performance minus the performance of the focal coordinate) in relation to ornament expression along with black or light blue arrows. The evolutionary pathway to the current state is biased toward right side because of intense sexual selection favoring long tails (i.e., actual evolutionary pathway depends on sexual selection in addition to viability selection due to flight cost and other costs, such as production costs of tail length and compensatory traits; see text). Narrow contour width indicates that small deviation from integrated, co-opted character sets had a strong negative effect on flight performance, as predicted by Norberg (1994). Here, for the illustrative purpose, I assumed that performance is determined by –4(*x* – 3)^2^ – 4(*y* – 3)^2^ + 7(*x* – 3)*(*y* – 3) where *x* denotes ornament expression whereas *y* denotes the expression of compensatory traits. The current state is determined here as the point in which the sum of performance surface and sexual selection surface (here, 5*x*) is maximized (see text for detailed explanation).

(See next page for Figure S3)

(Continued from the previous page)

**Figure S4.** A phylogenetic comparative experiment. Rather than reducing a large extent of ornamentation in a single species, which can result in overcompensation (light blue arrow), researchers should reduce small extents of ornamentation in multiple species with varying expressions of ornamentation, without being bothered by overcompensation (dark green arrow). Black arrow indicates a hypothetical evolutionary pathway to the current state of the most ornamented species. Less-ornamented species are assumed to be present somewhere near the evolutionary pathway (i.e., they are here assumed to be hypothetical ancestral states of the most ornamented species; sensu Matyjasiak et al. 2009). See Fig. 3 for detailed information (note that this figure is derived from the upper panel in Fig. 3). For easy understanding, dotted line is shown here as a ridge line, above which overcompensation occurs.
