# Supplementary material for: Compensatory traits can explain the concave cost function of purely sexual traits
Source: Ecol Evol. 2024 Jan 14;14(1):e10850. doi: 10.1002/ece3.10850 (PMC10788312; doi:10.1002/ece3.10850)
Supplement: Supplementary file 2 — Table S1 [file ECE3-14-e10850-s001.docx]

**Table S1** Data set for the current study

| Model ID | Treatment  (×10mm) | Absolute angle deviated from the horizontal plane |
| --- | --- | --- |
| 1 | 0 | 23.2 |
| 1 | -2 | 11.8 |
| 1 | -4 | 0.2 |
| 1 | -6 | 8.8 |
| 1 | -8 | 19.0 |
| 2 | 0 | 17.0 |
| 2 | -2 | 8.1 |
| 2 | -4 | 0.8 |
| 2 | -6 | 9.8 |
| 2 | -8 | 18.0 |
| 3 | 0 | 25.5 |
| 3 | -2 | 14.8 |
| 3 | -4 | 4.7 |
| 3 | -6 | 2.9 |
| 3 | -8 | 9.1 |
| 4 | 0 | 22.4 |
| 4 | -2 | 9.5 |
| 4 | -4 | 2.2 |
| 4 | -6 | 11.4 |
| 4 | -8 | 19.8 |

See text for detailed information
